# Telestroke Assessment With Perfusion CT Improves the Diagnostic Accuracy of Stroke vs. Mimic

**DOI:** 10.3389/fneur.2021.745673

**Published:** 2021-12-03

**Authors:** Lucinda Tran, Longting Lin, Neil Spratt, Andrew Bivard, Beng Lim Alvin Chew, James W. Evans, William O'Brien, Christopher Levi, Timothy Ang, Khaled Alanati, Elizabeth Pepper, Carlos Garcia-Esperon, Mark Parsons

**Affiliations:** ^1^Department of Neurology and Neurophysiology, Liverpool Hospital, Liverpool, NSW, Australia; ^2^South Western Sydney Clinical School, University of New South Wales, Liverpool, NSW, Australia; ^3^Department of Neurology, John Hunter Hospital, Newcastle, NSW, Australia; ^4^Brain and Mental Health Program, Hunter Medical Research Institute, Newcastle, NSW, Australia; ^5^School of Biomedical Sciences and Pharmacy, College of Health, Medicine and Wellbeing, University of Newcastle, Newcastle, NSW, Australia; ^6^Melbourne Brain Centre, University of Melbourne, Parkville, VIC, Australia; ^7^Neurosciences Department, Gosford Hospital, Gosford, NSW, Australia; ^8^Neurology Department, Royal Prince Alfred Hospital, Sydney, NSW, Australia; ^9^Neurology Department, Prince of Wales Hospital, Sydney, NSW, Australia; ^10^Ingham Institute for Applied Medical Research, Liverpool, NSW, Australia

**Keywords:** stroke, telestroke, imaging—computed tomography, transient ischaemic attack (TIA), CT perfusion (CTP), stroke mimic

## Abstract

**Background and Purpose:** CT perfusion (CTP) has been implemented widely in regional areas of Australia for telestroke assessment. The aim of this study was to determine if, as part of telestroke assessment, CTP provided added benefit to clinical features in distinguishing between strokes and mimic and between transient ischaemic attack (TIA) and mimic.

**Methods:** We retrospectively analysed 1,513 consecutively recruited patients referred to the Northern New South Wales Telestroke service, where CTP is performed as a part of telestroke assessment. Patients were classified based on the final diagnosis of stroke, TIA, or mimic. Multivariate regression models were used to determine factors that could be used to differentiate between stroke and mimic and between TIA and mimic.

**Results:** There were 693 strokes, 97 TIA, and 259 mimics included in the multivariate regression models. For the stroke vs. mimic model using symptoms only, the area under the curve (AUC) on the receiver operator curve (ROC) was 0.71 (95% CI 0.67–0.75). For the stroke vs. mimic model using the absence of ischaemic lesion on CTP in addition to clinical features, the AUC was 0.90 (95% CI 0.88–0.92). The multivariate regression model for predicting mimic from TIA using symptoms produced an AUC of 0.71 (95% CI 0.65–0.76). The addition of absence of an ischaemic lesion on CTP to clinical features for the TIA vs. mimic model had an AUC of 0.78 (95% CI 0.73–0.83)

**Conclusions:** In the telehealth setting, the absence of an ischaemic lesion on CTP adds to the diagnostic accuracy in distinguishing mimic from stroke, above that from clinical features.

## Introduction

Access to reperfusion therapy for stroke is largely related to the timely and accurate diagnosis of stroke (1). However, the availability of reperfusion therapy is still largely limited in rural areas of many countries, such as Australia (2). Telestroke with multimodal CT (mCT) imaging, which includes non-contrast CT brain, CT angiography (CTA), and CT perfusion (CTP), has been demonstrated to be an effective way of increasing thrombolysis in these regions and has also been used to select patients who are likely to benefit from transfer to a comprehensive stroke centre for endovascular thrombectomy (3).

Accurate diagnosis of stroke mimics from stroke and transient ischaemic attack (TIA) is required to avoid unnecessary treatment and transfer of patients in the telestroke setting. However, there has been little research into the accuracy of identifying mimics from ischaemic events in the telestroke setting. Most of the research differentiating ischaemic events from mimics has focused primarily on face-to-face clinical assessment in the Emergency Department (4–8). Common predictors of mimics include younger age, lower National Institutes of Health Stroke Scale (NIHSS), lack of stroke risk factors (such as atrial fibrillation and hypertension), lack of lateralising weakness, loss of consciousness, confusion, paraesthesia, or previous diagnosis of migraine or epilepsy (4–7, 9). There are fewer studies, which examine imaging factors to differentiate stroke from mimic. Chang et al. (7) assessed CTA in improving the diagnostic accuracy of strokes from mimics, and their logistic regression model using clinical features and evidence of atherosclerosis on CTA yielded an overall accuracy of 91.4%.

There have been a few studies looking at clinical predictors to develop risk prediction tools for distinguishing between stroke and mimic in the telestroke population (10–12). In a recent study by Tu et al. (13), which externally validated four existing stroke mimic prediction scales, the scale with the highest discrimination for stroke mimics had an area under the receiver operator curve value of 0.75. Similar to the studies which focused on face-to-face clinical evaluation in the Emergency Department, studies of telestroke assessment found that mimic patients had lower NIHSS scores, were younger in age, usually had a history of seizure, migraine or psychiatric illness, were less likely to have atrial fibrillation or hypertension, and had a lack of localising symptoms (10–12). However, none of these studies evaluated the utility of including imaging in predictive models.

The aim of the current study was to determine the clinical features that distinguish mimic from stroke and TIA in a telestroke setting, and whether mCT, in particular, CTP improves diagnostic accuracy.

## Materials and Methods

We retrospectively analysed consecutively recruited patients assessed by the Northern New South Wales Telestroke service from the time of introduction of telestroke to the area from April 2013 to March 2020. There were five spoke hospitals in total. The distance between the spoke sites and the hub ranged from 167 to 386 km. At each spoke hospital, cameras for consultation were provided, and training was provided to physicians for the use of the Face Arm Speech Time (FAST) scale (13).

Radiology technicians were trained to perform an mCT imaging protocol which included non-contrast brain CT, CTA, and CTP at baseline, with either non-contrast brain CT or MRI performed at 24–48 h. MIStar (Apollo Medical Imaging Technology) was used to process perfusion CT to generate cerebral blood volume, cerebral blood flow (CBF), mean transit time, delay time (DT) maps, summary ischaemic core, and penumbra maps. Tissue with DT >3 sec and relative CBF >30% of normal tissue were defined as the penumbra, and tissue with DT >3 sec and relative CBF <30% of the contralateral hemisphere were defined as the ischaemic core. More detailed information on the imaging protocol has been previously published (3).

The criteria for stroke call were defined as FAST scale positive neurological symptoms presenting within 4.5 h of symptom onset from April 2013 to November 2017, after which the time window was expanded to 24 h given the findings of the Diabetes Autoimmunity Withdrawn in New Onset Patients (DWI or CTP Assessment with Clinical Mismatch in the Triage of Wake-UP and Late Presenting Strokes Undergoing Neurointervention with Trevo) trial (14). The Northern New South Wales Telestroke service is staffed by stroke neurologists from the John Hunter and Gosford District Hospitals and was responsible for clinical telestroke assessment and interpreting the mCT scans in the acute phase.

The following patient characteristics were collected: demographic data (age and sex), the record of symptoms (hemiparesis, dysphasia, visual disturbance, paraesthesia, isolated facial palsy, isolated limb weakness, dysarthria, ataxia, dizziness, decreased level of consciousness, disorientation, headache, seizure, and non-localising symptoms), and NIHSS score at presentation. Imaging findings, such as the presence of vessel occlusion on CTA, volume of automated CTP lesions (core and penumbra), and follow-up imaging results, were also recorded. The final diagnosis was reviewed by a telestroke neurologist and based upon all clinical and imaging findings, such as subsequent imaging and clinical assessments. Stroke was defined as symptoms consistent with a stroke syndrome with the presence of infarction on follow-up imaging or persisting beyond 24 h. TIA was defined as typical stroke symptoms lasting <24 h with no infarction on follow-up imaging. Mimics were defined as an atypical stroke presentation with the absence of infarction on follow-up CT or MRI, and/or if symptoms were explained by a clinically determined alternative aetiology (excluding intracranial haemorrhage). Uncertain cases were decided by consensus between two stroke neurologists. Patients who did not have acute CTP were excluded from this study. Ethics approval was obtained from the Hunter New England Human Research Ethics Committee (HNEHREC reference no: 2019/ETH13062).

Patient characteristics were summarised by percentage and group differences and were tested by Pearson Chi-squared test. Logistic regression was performed to assess the significance of patient characteristics in differentiating mimic from stroke, followed by receiver operation characteristic (ROC) curve analysis with the area under curve (AUC) indicating diagnostic accuracy. Five models were constructed to examine the diagnostic accuracy of using clinical features only and then with the addition of imaging findings to differentiate the additional benefit in diagnostic accuracy with CTA and/or CTP. The core volume on the summary CTP maps was used as the CTP ischaemic lesion volume in the statistical analysis. Only the symptoms, which were significant as a univariate predictor, were included in the models. Please see Table 1 for further detail regarding the models and the variables included as predictors. Similarly, logistic regression and ROC analysis were also performed to assess the diagnostic accuracy in differentiating mimic from TIA, and five analogous models were also created (Table 1). All statistical analysis was done using STATA 13.0 (Stata Corp, College Station, TX, USA), with CI set at 95% and a significant level set at 0.05.

**Table 1:** Predictors used in logistic regression models for stroke vs. mimic and transient ischaemic attack vs. mimic.

**Stroke vs. Mimic**	
Model	Predictors
A1	Symptoms
A2	Symptoms, NIHSS score, age, sex
A3	Symptoms, NIHSS score, age, sex, CTA (no occlusion)
A4	Symptoms, NIHSS score, age, sex, CTP (ischaemic lesion of 0 mL)
A5	Symptoms, NIHSS score, age, sex, CTA (no occlusion), CTP (ischaemic lesion of 0 mL)
**TIA vs. Mimic**	
Model	Predictors
B1	Symptoms
B2	Symptoms, NIHSS score, age, sex
B3	Symptoms, NIHSS score, age, sex, CTA (no occlusion)
B4	Symptoms, NIHSS score, age, sex, CTP (ischaemic lesion of 0 mL)
B5	Symptoms, NIHSS score, age, sex, CTA (no occlusion), CTP (ischaemic lesion of 0 mL)

*NIHSS, National Institutes of Health Stroke Scale; CTA, computed tomography angiography; CTP, computed tomography perfusion; TIA, transient ischaemic attack*.

## Results

In total, 1,513 patients were assessed over this time period by the Northern New South Wales Telestroke service, and 1,074 patients assessed had CTP and were included in this study. The most common reasons for not proceeding with mCT included evidence of established stroke, bleed, or tumour on non-contrast CT brain (121 patients, 28%), minor symptoms (119, 27%), or presentation deemed unlikely to be stroke (79 patients, 18%). Stroke was diagnosed in 693 (64%) patients, 97 patients (9%) were diagnosed with TIA, 259 (24%) patients were classified as mimics, and 25 (2%) patients were found to have an intracranial bleed. The diagnosis of stroke was confirmed on MRI imaging in 288 (42%) patients, 326 (47%) cases were confirmed on CT imaging, and 79 (11%) cases were clinical diagnoses. With regards to the aetiology of the cases classified as a mimic, there were 47 (18%) seizures, 28 (11%) were diagnosed with a functional neurological syndrome, 25 (10%) with syncope or hypotension, 22 (8%) with migraine, 22 (8%) with delirium or metabolic encephalopathy, 20 (8%) with peripheral vestibulopathy, 11 (4%) with infection, 10 (4%) with peripheral nerve lesion, 10 (4%) with drug intoxication or medication side effect, and 7 (3%) with intracranial tumour. There were 28 (11%) cases of mimics that had an identified cause, but <5 cases for each cause, and 29 (11%) cases where the aetiology of the mimic could not be further determined.

There were considerable differences between the stroke and mimic groups (Table 2). In terms of demographics, those with stroke tended to be older than the stroke mimics, with 73% over age 65 in the stroke group compared to 50% in the mimics (*p* < 0.001). There was a higher proportion of male patients in the stroke group than the mimic group (64 vs. 44%, *p* < 0.001). The mimic group had lower NIHSS scores, 65% having an NIHSS score ≤ 4 in the mimic group vs 42% in the stroke group (*p* < 0.001). Hemiparesis, aphasia, the visual disturbance were more common in the stroke group compared to the mimic group (Table 2). Clinical features, which were more common in the mimic group, were vertigo, decreased level of consciousness, non-localising symptoms, disorientation, headache, and seizures (Table 2). The symptom of paraesthesia did not differ between the two groups (Table 2).

**Table 2:** Predictors for stroke vs. mimic and TIA vs. mimic.

	**Stroke** **(*n* = 693)**	**Mimic** **(*n* = 259)**	**TIA** **(*n* = 97)**	**P value** **for stroke** **vs. mimic**	**P value** **for TIA** **vs. mimic**
CTP ischaemic lesion = 0 ml	246 35.5%	245 94.6%	86 88.7%	<0.001	0.051
Age>65	510 73.6%	129 49.8%	64 65.9%	<0.001	0.007
Male	449 64.8%	115 44.6%	51 52.6%	<0.001	0.178
Baseline NIHSS score ≤ 4	296 42.9%	164 65.1%	83 85.6%	<0.001	<0.001
***Clinical feature***					
Hemiparesis	444 64.1%	102 40.2%	54 56.3%	<0.001	0.007
Aphasia	222 32.0%	57 22.4%	N/A	0.004	N/A
Dysarthria	N/A	20 7.9%	17 17.7%	N/A	0.008
Visual disturbance	110 15.9%	20 7.9%	N/A	0.002	N/A
Paraesthesia	36 5.2%	21 8.3%	17 17.7%	0.078	0.011
Vertigo	12 1.7%	22 8.7%	3 3.1%	<0.001	0.073
Decreased level of consciousness	17 2.5%	35 13.8%	1 1.0%	<0.001	<0.001
Non-localising symptoms	6 0.9%	14 5.5%	1 1.0%	<0.001	0.065
Disorientation	12 1.7%	19 7.5%	0 0%	<0.001	0.006
Headache	6 0.9%	19 7.5%	N/A	<0.001	N/A
Seizure	4 0.6%	12 4.7%	0 0%	<0.001	0.03

*TIA, transient ischaemic attack; CTP, computed tomography perfusion, NIHSS, National Institutes of Health Stroke Scale*.

**Figure 1:**
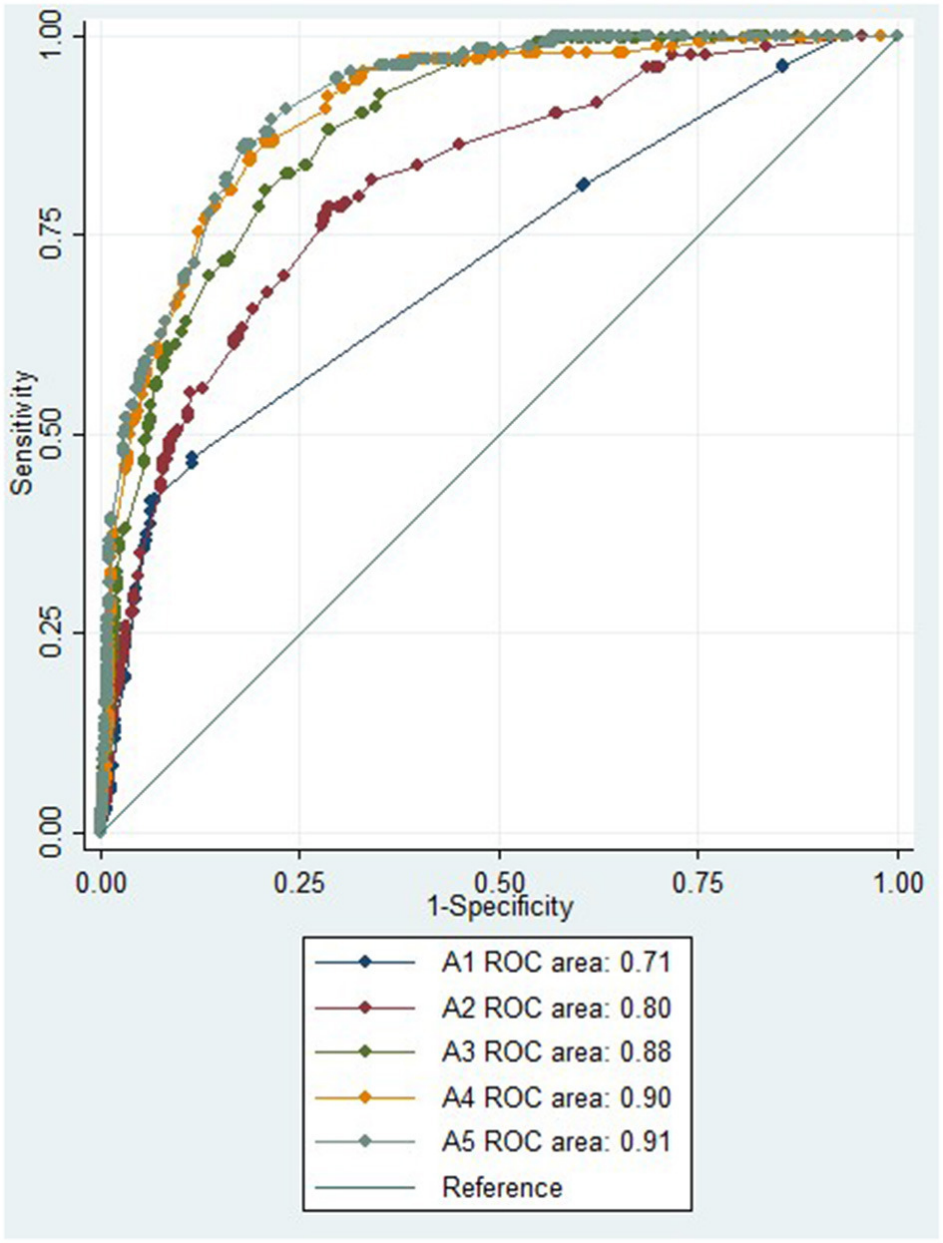
ROC for multiple regression model to distinguish between stroke and mimic. ROC, receiver operator curve; NIHSS, National Institutes of Health Stroke Scale; CTA, computed tomography angiography; CTP, computed tomography perfusion.

The diagnostic accuracy of an absent CTP core/penumbra lesion in a univariate regression model for distinguishing stroke from stroke mimic produced an AUC of 0.79 (95% CI 0.77–0.81), and this was higher than the diagnostic accuracy of no vessel occlusion on CTA, which had an AUC of 0.72 (95% CI 0.70–0.74). Multivariate regression models for differentiating stroke from mimics were created using symptoms that were significant in univariate analysis (Table 2), acute NIHSS score, age, sex, and CT imaging findings (Table 3). The first multivariate regression model, A1, using symptoms only, produced an AUC of 0.71 (95% CI 0.67–0.75; Figure 1). In the second model, A2, with the addition of acute NIHSS score, age, and sex to the initial model, the AUC improved to 0.80 (95% CI 0.77–0.84). In the third model, A3, with the addition of the absence of CTA occlusion to the same clinical features used in model A2, the AUC was improved to 0.88 (95% CI 0.86–0.90). In the fourth model, A4, with the addition of an absent CTP core/penumbra lesion to clinical features, the AUC was improved to 0.90 (95% CI 0.88–0.92). In the fifth model, A5, the addition of both absence of occlusion on CTA and absence of CTP core/penumbra lesion yielded the model with the highest diagnostic accuracy, AUC of 0.91 (95% CI 0.89–0.93).

**Table 3:** Multivariate regression model for predicting mimic vs. stroke.

**Mimic vs. stroke**	**Odds ratio**	***P* value**	**95% CI**	
CTP lesion = 0 ml	31.28	<0.001	16.43	59.54
Age > 65	0.33	<0.001	0.22	0.50
Male	0.40	<0.001	0.27	0.59
NIHSS score ≤ 4	1.53	0.048	1.00	2.33
Aphasia	1.35	0.207	0.84	2.16
Visual disturbance	0.92	0.826	0.46	1.84
Paraesthesia	0.82	0.576	0.41	1.64
Vertigo	4.67	0.001	1.82	11.98
Decreased LOC	12.82	<0.001	5.04	32.58
Non-localising symptoms	6.61	0.007	1.67	26.09
Disorientation	7.62	<0.001	2.90	19.99
Headache	4.17	0.017	1.29	13.49
Seizure	9.57	0.004	2.07	44.09

*CTP, computed tomography perfusion; NIHSS, National Institutes of Health Stroke Scale; CI, confidence interval*.

With regards to the differentiating between TIA and mimics, there was no significant difference between the two groups in terms of sex (Table 2). Older age, lower NIHSS scores (≤ 4), hemiparesis, dysarthria, and paraesthesia were more common in those with TIA compared to mimic (Table 2). Decreased levels of consciousness, disorientation, and seizure were more likely to be present in mimics than in patients with TIA (Table 2). Other individual clinical features, such as vertigo, and non-localising symptoms, did not distinguish between mimic and TIA (Table 2).

**Figure 2:**
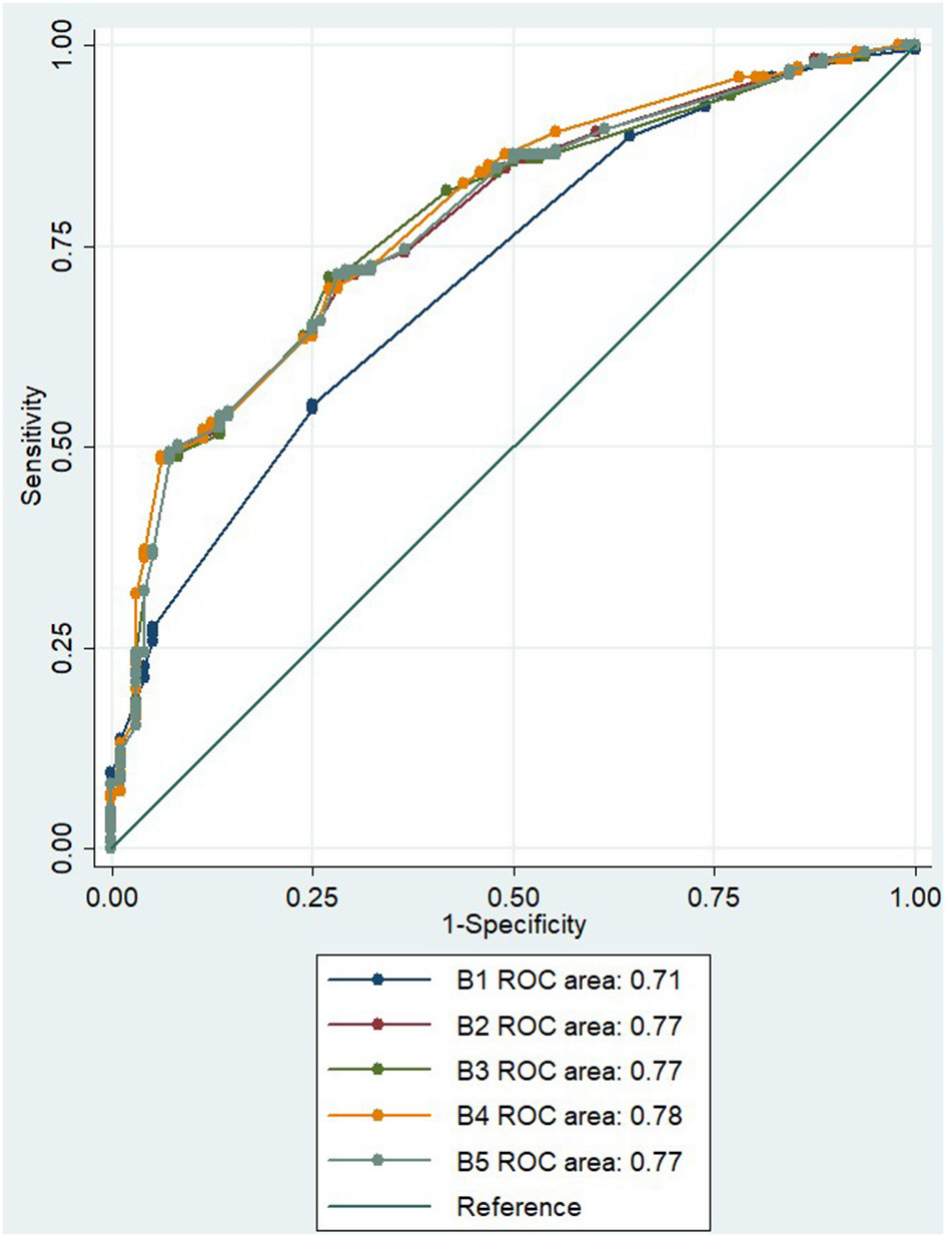
ROC for multiple regression model to distinguish between TIA and mimic. ROC, receiver operator curve; NIHSS, National Institutes of Health Stroke Scale; CTA, computed tomography angiography; CTP, computed tomography perfusion.

In the initial multivariate regression model for distinguishing mimic from TIA using symptoms only (B1), the AUC was 0.71 (95% CI 0.65–0.76; Figure 2). The AUC was improved to 0.77 (95% CI 0.72–0.82) when age and NIHSS score were added to the model (B2). With the addition of no occlusive lesion on CTA to the model (B3), the AUC did not improve, AUC 0.77 (95% CI 0.72–0.83; Figure 2). Similarly, with the addition of absence of CTP lesion in the B4 model, the AUC did not significantly improve, AUC 0.78 (95% CI 0.73–0.83; Figure 2), even though the absence of a CTP lesion was a borderline significant independent variable in the multivariate equation (*p* = 0.054, Table 4). The addition of both CTA and CTP findings of no lesion in model B5 has a similar AUC of 0.77 (95% CI 0.72–0.82).

**Table 4:** Multivariate regression model for predicting mimics vs. TIA.

**Mimics vs. TIA**	**Odds**	***P***	**95% CI**	
CTP lesion = 0 ml	2.84	0.054	0.98	8.22
Age > 65	0.33	<0.001	0.18	0.59
NIHSS score ≤ 4	0.34	0.003	0.16	0.70
Hemiparesis	0.58	0.065	0.33	1.03
Dysarthria	0.29	0.007	0.12	0.71
Vertigo	4.21	0.031	1.14	15.53
Paraesthesia	0.33	0.009	0.15	0.76
Decreased LOC	15.22	0.009	1.95	118.43
Non-localising symptoms	2.60	0.386	0.29	22.58
Disorientation	1	(omitted)		
Seizure	1	(omitted)		

*TIA, transient ischaemic attack; CTP, computed tomography perfusion, NIHSS, National Institutes of Health Stroke Scale; CI, confidence interval*.

## Discussion

The results are consistent with previous studies in non-telestroke and telestroke populations showing that there are a number of clinical features that can help differentiate between stroke and mimics (4, 6, 11). Patients with stroke tended to be older, have lateralising symptoms, and have higher NIHSS scores compared to mimics (4, 6, 11). In contrast, symptoms which occurred more commonly in mimic, such as vertigo, decreased level of consciousness, confusion, headache, and seizure, were less likely to be strokes and tended to be the common presentation of mimic conditions, such as migraine, seizures, and metabolic disturbances (11). The diagnostic accuracy of our model using clinical features to differentiate between stroke and mimic was similar to that of Ali et al. (12) who had a result of 0.72, using age, NIHSS, history of atrial fibrillation, hypertension, and facial weakness as predictors in their model. Similar to other studies looking at the primary presentation of stroke, the rate of mimics was about 24% (4, 5, 8, 11).

Regression analysis to distinguish between stroke and mimic showed that the presence of a CTP lesion in isolation was as accurate as clinical features in diagnosis. This is particularly important in a telestroke setting, where it can be difficult to appreciate nuances in clinical features remotely. When adding the absence of a CTP lesion to the clinical features, the ROC area of 0.9 was extremely high. Whilst there are no comparable studies using a telehealth population and CTP in their regression models, the accuracy of our model is as least as accurate as studies where face-to-face clinical assessment occurred and included CTA findings (but not CTP) in their models (6). Our model using CTP in addition to clinical features is considerably more accurate than that of previous stroke mimic prediction scales used in the telestroke context, which have only included clinical features (12). Our findings support the use of CTP in improving diagnostic confidence when attempting to distinguish stroke from mimics in the telehealth setting.

With regards to differentiating between TIA and mimic, as expected, decreased level of consciousness was more likely to occur in mimic than TIA, given that decreased level of consciousness is more commonly associated with metabolic disturbances and seizures than ischaemic pathology (7). Interestingly, lower NIHSS scores were more common in those with TIA compared to mimic. One possible explanation of this finding is that the conditions which mimic stroke can present with generalised weakness which may be scored highly on the NIHSS, compared to TIA which usually presents with minor or rapidly resolving focal neurological symptoms. Whilst not as many of the symptoms were statistically significant to differentiate between mimics and TIA individually in comparison to the stroke vs. mimic model, collectively, as demonstrated in the multivariate regression model, the AUC was 0.71 and comparable to that using symptoms alone for distinguishing between stroke and mimic. This model is comparable to the AUC of the Dawson Model and slightly less than the diagnosis of TIA (DOT) model previously described to differentiate between TIA and TIA mimics (7).

The use of mCT did not improve the diagnostic accuracy of differentiating between TIA and mimic in this study and this could be due to limited power from the small number of patients diagnosed with TIA (9%) and the possibility of misclassification in the final diagnosis between those in the TIA and mimic groups given the inherent difficulties to accurately and reliably apply the operationalised definitions. Interestingly, ischaemic core/penumbra lesions were seen in a subset of patients with TIA, and there was a strong trend for the absence of core/penumbra lesion to predict mimic from TIA in multivariate analysis (Table 4). It was not expected that the absence of an ischaemic lesion on automated CTP maps would be useful to differentiate between TIA and mimic, because neither conditions are expected to have a core/penumbra lesion on CTP. This raises the possibility that assessment of the individual perfusion maps might also assist in distinguishing between mimic and TIA (15–17). This potentially provides an avenue of further investigation to aid the clinically difficult task of differentiating TIA from mimic, especially via telemedicine.

A limitation of the current study is its retrospective analysis which exposes it to the possibility of information bias. There is a small proportion of patients diagnosed with stroke clinically who did not have subsequent imaging to confirm the diagnosis. It is possible that a subset of these patients were mimics who were incorrectly classified. To improve the external validity of the study, patients referred to the telestroke service but who did not proceed to have CTP were excluded from the analysis to reflect the clinical practice that patients diagnosed with the established stroke, bleed, or tumour on non-contrast CT brain, or minor symptoms, are generally ineligible for reperfusion therapy. We limited our analysis to the patients presenting with stroke-like symptoms being considered for reperfusion therapy, to provide evidence whether CTP could be used as a tool to improve diagnostic confidence between strokes and mimic. However, selecting this particular population and excluding those with minor symptoms or a syndrome clinically inconsistent with stroke may have potentially affected the analysis of differentiating TIA vs. mimics.

The results of this study support the use of CTP to improve the accuracy of differentiating between stroke and mimics in a telehealth setting for patients being considered for reperfusion therapy. Whilst it is generally not harmful to give thrombolysis to mimics (18), when one does, it can falsely elevate rates of good outcome (as the mimics generally have an excellent natural history). Further, the cost-effectiveness of using a relatively expensive medication and more intensive (and expensive) post-lysis inpatient care, unnecessary investigations, and/or patient transfer has yet to be properly assessed. The feasibility of using CTP in rural areas with expert stroke input via telehealth for improving access to stroke-specific therapy, such as thrombolysis, has been demonstrated in a previous study with a similar population (3).

In summary, patients presenting with an acute stroke-like presentation assessed via telemedicine for reperfusion therapy, such as acute CTP, can help reliably distinguish strokes from mimics.

## Data Availability Statement

The raw data supporting the conclusions of this article will be made available by the authors, without undue reservation.

## Ethics Statement

The studies involving human participants were reviewed and approved by Hunter New England Human Research Ethics Committee. Written informed consent for participation was not required for this study in accordance with the national legislation and the institutional requirements. An opt-out consent form was available for participants who did not wish to participate.

## Northern NSW Telestroke Investigators

Ms. Rachel Peake, Dr. James Hughes, Dr. Lisa Dark, Dr. Nick Ryan, Dr. Matt Shepherd, Dr. Osama Ali, Dr. Hugh Reid, Ms. Fiona Minett, Ms. Jaclyn Birnie, Ms. Amanda Buzio, Dr. Iain Bruce, Dr. Alan Tankel, Ms. Kim Parrey, Dr. Matthew Kinchington, Dr. Ferdinand Miteff, Dr. Pablo Garcia Bermejo, Dr. Andre Loiselle, Dr. James Thomas, Dr. Jessica Stabler, Dr. Mohammad Amin, Ms. Michelle Russell, Ms. Angela Royan, Mr. Brett Roworth, and Dr. Mary Morgan.

## Author Contributions

CG-E, BC, LT, and LL organised the database. LL performed the statistical analysis and wrote sections of the manuscript. LT and MP wrote the initial draft of the manuscript. All authors contributed to the revision of the manuscript, approved the final manuscript for publication, contributed to the conceptualisation, and design of the study.

## Funding

This project was supported by the Australian Government's Medical Research Future Fund (MRFF) as part of the Rapid Applied Research Translation Program.

## Conflict of Interest

The authors declare that the research was conducted in the absence of any commercial or financial relationships that could be construed as a potential conflict of interest. The reviewer BY declared a shared affiliation, with no collaboration, with one of the authors AB to the handling editor at the time of the review.

## Publisher's Note

All claims expressed in this article are solely those of the authors and do not necessarily represent those of their affiliated organizations, or those of the publisher, the editors and the reviewers. Any product that may be evaluated in this article, or claim that may be made by its manufacturer, is not guaranteed or endorsed by the publisher.
